# Coronary artery calcium and cardiovascular risk factors analysis after radiotherapy for breast cancer (the CLARIFIER: a gender-based preventive medicine study)

**DOI:** 10.3389/fcvm.2025.1615793

**Published:** 2025-08-04

**Authors:** Daniela Trabattoni, Maria Cristina Leonardi, Maria Elisabetta Mancini, Barbara A. Jereczek-Fossa, Federica Cattani, Giulia Santagostino Baldi, Alice Bonomi, Arianna Galotta, Saima Mushtaq, Andrea Annoni, Davide Alio, Maria Giulia Vincini, Cristiana Iuliana Fodor, Ludovico La Grutta, Piero Montorsi, Gianluca Pontone

**Affiliations:** ^1^ Centro Cardiologico Monzino, IRCCS, Milan, Italy; ^2^ Division of Radiation Oncology, European Institute of Oncology (IEO), IRCCS, Milan, Italy; ^3^ Department of Oncology and Hemato-Oncology, University of Milan, Milan, Italy; ^4^ Unit of Medical Physics, European Institute of Oncology (IEO), IRCCS, Milan, Italy; ^5^ Biomedicine, Neuroscience and Advanced Diagnostics (BiND) Department, University of Palermo, Palermo, Italy; ^6^ Department of Clinical Sciences and Community Health, University of Milan, Milan, Italy; ^7^ Department of Biomedical, Surgical and Dental Sciences, University of Milan, Milan, Italy

**Keywords:** breast cancer, radiation therapy, CV risks, coronary artery calcium, coronary disease, prevention

## Abstract

**Introduction:**

Patients receiving thoracic radiation (RT) are at increased risk for heart disease. Coronary artery calcium (CAC) is an independent risk factor for cardiac events.

**Aim:**

The aim of this prospective, joint-institution, study was to analyze the relationship between cardiovascular risk factors (CVRF) known before breast cancer diagnosis and treatment, and the risk of developing coronary events in women undergoing adjuvant breast radiotherapy by measuring CAC.

**Methods:**

Women (*n* = 92) diagnosed with early-stage breast cancer between 2010 and 2016 were enrolled and underwent cardiologic clinical assessment and coronary CT-scan for CAC score analysis, at least 5 years after RT.

**Results:**

Data obtained from 91/92 patients, showed a 36.2% incidence of pathologic Agatston CAC score, independent of the irradiated breast side. After grouping patients according to the total number of CVRF [group 1, *n* = 55 (60.4%): 0–2 CV risk factors; group 2, *n* = 36 (39.6%): 3–5 CV risk factors] significant differences were observed in CAC scores. Normal CAC scores (Agatston 0) were recorded in 70.9% in group 1 vs. 41.7% in group 2 (*p* = 0.005), while CAC-3 (Agatston ≥ 300) in 11.1% of group 2 only (*p* = 0.02), corresponding to clinical evidence of coronary disease. The risk of cardiac events was associated with increased age, early menopause, hypertension, high cholesterol levels, and smoking habits at the time of RT.

**Conclusion:**

This study helps to identify women at high-risk for cardiovascular events before RT and implement the best possible prevention of late post cancer treatment events.

**Clinical Trial Registration:**

ClinicalTrials.gov, Identifier (NCT05775822).

## Introduction

The American Society of Clinical Oncology (ASCO) 2017 guidelines on cardiac monitoring during cancer treatments identified patients receiving thoracic radiotherapy (RT) ≥30 Gy (with the heart in the field) as being at increased risk for developing radiation-induced heart disease. Therefore, an active screening of baseline modifiable cardiac risk factors and therapy-induced cardiotoxicity in this high-risk population (1) is strongly suggested.

Over the past 15 years, a body of evidence from studies comparing left- with right-sided hemithorax RT has described the relationship between RT for breast cancer and the risk of subsequent heart disease. Since the heart is located on the left side of the chest, the radiation dose to the heart tends to be larger for left-sided than for right-sided breast cancer. An increased risk of heart disease associated with left-sided compared with right-sided RT has been interpreted as evidence of radiation-induced heart disease. However, several studies have yielded mixed results, with some supporting (2, 3) and others disputing (4, 5) the association between breast radiotherapy and increased risk of heart disease.

There is, however, a large inter-patient variation in the radiation dose to the heart, in particular for left-sided treatments. Therefore, a more precise measurement of the dose to the cardiac structures is necessary to relate it to a later risk of heart disease. In 2013, it was reported that the risk of major coronary events increased linearly by 7.4% per Gy (*p* < 0.001) with the mean radiation dose to the heart, with no apparent threshold (6). While the incidental dose to the heart is strongly related to cardiac events, other risk factors can contribute to the excess risk, such as advanced age, history of cardiovascular diseases, diabetes, smoking, and high BMI. The current study focused on coronary artery calcium (CAC), which is an independent risk factor for adverse cardiac events that can be mitigated with preventive medical therapy. This study aims to analyze the CAC score at a mean time of 5 years after RT and the incidence of CV events on the long-term follow-up after RT to disclose any correlation between CV risk factors present before RT and coronary disease development on FU, after adjusting for confounding factors (mean heart dose).

## Materials and methods

**Figure 1:**
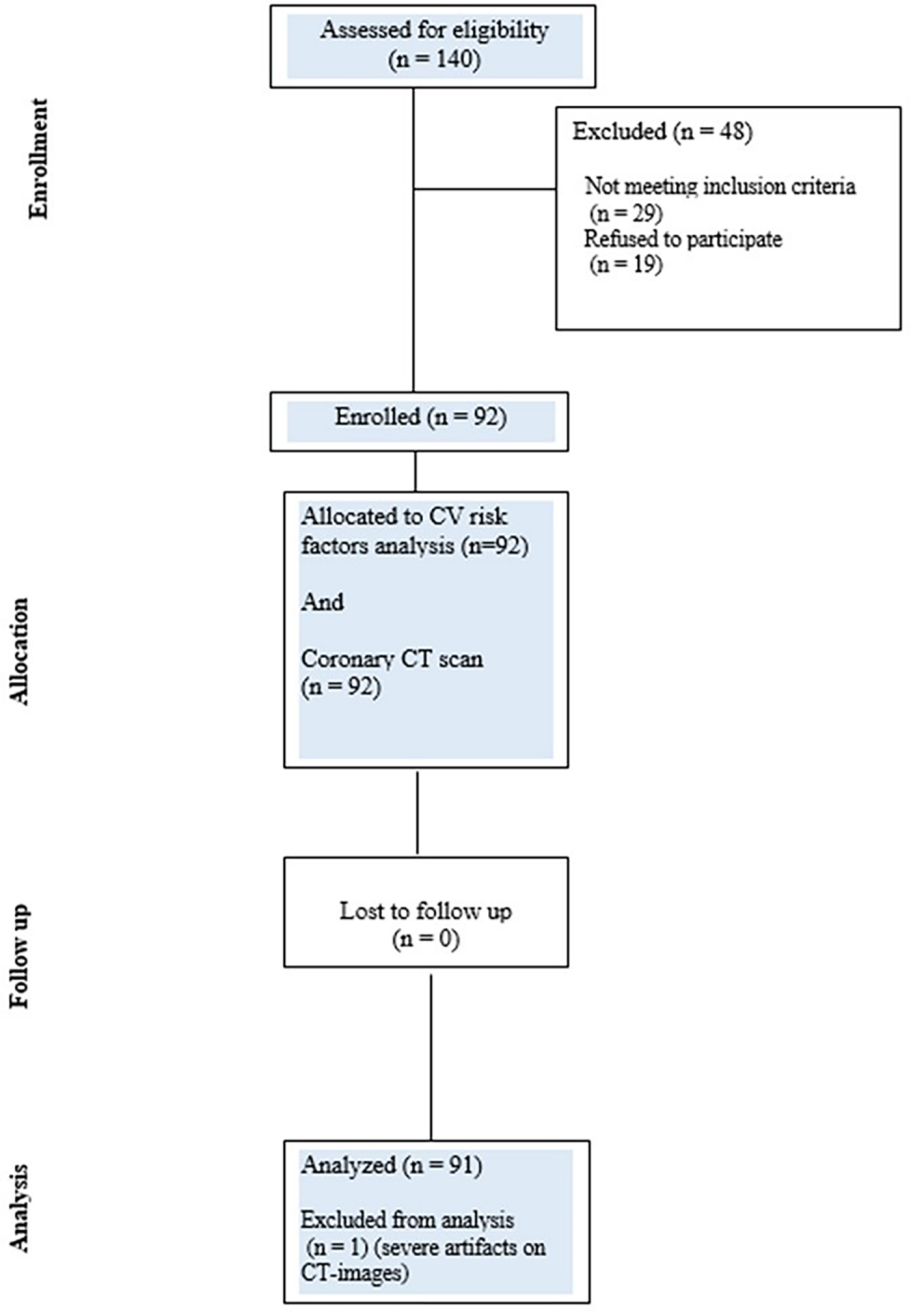
CONSORT diagram showing the flow of participants through each stage of the CLARIFIER study.

This is an interventional study conducted as part of a collaborative effort by the European Institute of Oncology and Centro Cardiologico Monzino, Milan, Italy. Between February 2011 and July 2016, 358 consecutive patients affected by breast cancer and treated with breast conservative treatment at the European Institute of Oncology were selected from a dedicated database maintained in the Radiotherapy Division. Of these patients, 140 were willing to participate in the research project. To be included in the study, the patients had to fulfill the following criteria: a diagnosis and treatment within the period 2010–2016, an age younger than 65 years at the time of radiotherapy, unilateral breast-conserving surgery for breast cancer with known laterality, no prior diagnosis of invasive cancer (except for non-melanoma skin cancer), and no previous thoracic radiotherapy. Out of 140 patients, 48 were excluded for failing to meet the eligibility criteria (*N* = 29) and for refusal after being fully informed on the study (*N* = 19) (Figure 1), leaving 92 subjects available for analysis. All patients gave a written consent for the study, which was approved by the Ethics Committee of the Centro Cardiologico Monzino (CCM1505-RE3159) on 27 July 2021.

### Radiotherapy details

All women received whole breast RT either with the three-dimensional conformal (3DCRT) technique using tangential fields (x-ray with energy of 6/18 MV) or with intensity-modulated radiotherapy (IMRT) using tomotherapy in direct modality (6 MV x-rays). Two schedules of moderate hypofractionation were applied: 45 Gy to the whole breast plus a 5 Gy concomitant boost in 20 fractions over 4 weeks and 40.05 Gy to the whole breast plus a 48 Gy simultaneous integrated boost in 15 fractions over 3 weeks. At the time of the study, the mean dose to the heart and, later, the mean dose to the LAD were the only two constraints considered in treatment planning. The mean heart dose was based on the constraint recommended by the RTOG 1005 study (7) (mean dose <3.2 Gy for hypofractionation). This constraint, as well as the one used for the LAD (average mean LAD dose <10 Gy), fell within the range described in the literature (6, 8). None of the patients were treated with specific cardiac-sparing techniques (breath-hold, prone position).

CT simulation scans of patients lying in the supine position on a breast board with both arms raised above the head were retrieved. The whole heart was systematically contoured at the time of the initial treatment planning, while the left anterior descending artery was routinely delineated starting in 2015. For the purpose of the study, additional cardiac structures were segmented: the left ventricle (introduced into the contouring routine in 2023), the right coronary artery, and the circumflex coronary artery. To minimize interobserver variability, a single radiation oncologist performed the contouring task after receiving brief training from the Centro Cardiologico Monzino team.

All segmentations underwent subsequent review and validation under the direct supervision of a radiologist with >5 years of dedicated experience in cardiovascular imaging. This expert control was integral to ensuring anatomical accuracy and consistency across all cases.

The treatment was replanned for each patient. For each cardiac structure of interest, the dose constraints were chosen based on those most commonly used in the literature: mean dose to the whole heart; mean dose, V5Gy, and V23Gy to the left ventricle; mean dose, V30Gy, and V40Gy to the left anterior descending artery; and mean dose and maximum dose to the circumflex coronary artery and the right coronary artery. All these cardiac substructures, except for the whole heart and the LAD, were delineated, and the dose was recalculated for the purpose of the study, to provide a comprehensive picture of the dose contribution to cardiac injury.

### Calcium score assessment and risk profile groupings

Patients with >5 years of clinical follow-up have been enrolled in the Women Heart Center for a preventive clinical cardiology assessment visit, aimed at individualized risk profile assessment and a chest CT scan. All CT examinations were performed using a 256-slice-wide volume coverage CT scanner (Revolution CT; GE HealthCare, Milwaukee, WI, USA). Non-enhanced ECG-gated chest CT scans were conducted according to the recommendations of the Society of Cardiovascular Computed Tomography (SCCT) (9) and were centrally reported by a level III EACVI-certified reader (10–12). The following scan parameters were used: peak tube voltage, 120 kV; detector collimation, 160 mm using 256 rows by 0.625 mm on *z*-axis; detector geometry, 256 rows by 832 detection elements per row; high-contrast spatial resolution, 0.23 mm; slice thickness, 0.625; gantry rotation time, 280 ms; and prospective triggering. A body mass index (BMI)-adapted protocol was used for the tube current with the following parameters: for a BMI ≤ 26 kg m^−2^, 500 mA; for a BMI of 27–30 kg m^−2^, 600 mA; and for a BMI > 30 kg m^−2^, 650 mA.

Coronary artery calcium is commonly defined as a hyper-attenuating lesion >130 Hounsfield units (HU) of more than three pixels. The quantification of CAC was performed according to the Agatston score by multiplying the total CAC area in mm^2^ by a density factor ranging from 1 to 4 (1 for lesions with a density of 130–199 HU; 2 for lesions with a density of 200–299 HU; 3 for lesions with a density of 300–399 HU; 4 for densities ≥400 HU). Coronary Artery Calcium Data and Reporting System (CAC-DRS) (13) is a structured reporting scheme for all non-contrast CT scans in the evaluation of coronary artery disease (CAD).

The cumulative CAC score was calculated in accordance with the methods previously described by Agatston et al. (14). The patients were then categorized into four groups: 0 (very low), 1–99 (mild), 100–299 (moderate), and >300 (severe).

Information on systemic therapy, concomitant and previous disorders, body mass index, and lifestyle data (physical activity, diet, tobacco smoking, alcohol consumption, etc.) was also collected.

### Statistical analysis

The calculation of the sample size was based on the primary endpoint. A sample of 92 patients was identified as necessary to obtain a statistical power of 80% to detect a significant (*p* < 0.01, considering the Bonferroni correction for multiple tests) correlation coefficient of 0.35 (adjusted for four variables confounding) between the change in calcium score and any of the five major CV risk factors (arterial hypertension, smoking, dyslipidemia, diabetes mellitus, family history) measured at baseline. The sample will be increased to a total of 100 patients, considering a follow-up dropout of 10%.

The association between CAC score and CV risk factors was studied by multiple linear regression analysis, also adjusting for radiation dose delivered, age, and laterality. All tests were two-sided, and a *p*-value of <0.05 was considered statistically significant. Statistical analyses were performed with SAS software, version 9.4 (SAS Institute, Cary, NC, USA). Continuous variables were summarized using mean ± standard deviation (SD) if normally distributed, otherwise as median and interquartile range (IQR). Categorical variables were represented using frequencies and percentages. Continuous variables were compared using Student's *t*-test for independent samples or Mann–Whitney *U* test, according to the distribution; Chi-square test or Fisher's exact test was performed to compare categorical data, as appropriate. Correlations were assessed by using Spearman's correlation coefficients. The CAC score was log-transformed for analysis. Correlation was assessed using either Pearson or Spearman correlation coefficients, as appropriate. The association between CAC score and CV risk factors was studied by multiple linear regression analysis, also adjusting for radiation dose delivered, age, and laterality. All tests were two-sided, and a *p*-value of <0.05 was considered statistically significant. Statistical analyses were performed with SAS software, version 9.4 (SAS Institute, Cary, NC, USA).

## Results

With a mean follow-up of 8.8 ± 2 years after RT, 92 patients (mean age 56.8 ± 6.8 years were screened for baseline characteristics and cardiovascular risk factors (CVRF) (Tables 1, 2). They underwent a CT scan to assess CAC distribution and patterns according to CAC-DRS (13).

**Table 1:** Baseline demographic and clinical characteristics.

Baseline demographic and clinical characteristics	Women enrolled (*N* = 92)
Age (years ± SD)	56.6 ± 6.9
Family Hx for CAD	8 (8.8)
Hypertension (*n*, %)	22 (24.2)
Diabetes (*n*, %)	4 (4.4)
Dyslipidemia (*n*, %)	33 (36.2)
Smoking (*n*, %)	9 (9.9)
Overweight (*n*, %)	10 (11)
BMI (mean ± SD)	24.32 ± 4.36
Menopause at time of RT (*n*, %)	85 (93.4)
Age at menopause (years ± SD)	48 ± 5.2
Previous TIA or stroke (*n*, %)	1 (1)
Ischemic heart disease (*n*, %)	4 (4.4)
Peripheral vascular disease (*n*, %)	1 (1)
Arrhythmias (*n*, %)	4 (4.4)
Coronary artery disease (previous PCI or CABG) (*n*, %)	0 (0)
Heart failure (*n*, %)	0 (0)
Pericarditis/myocarditis	0 (0)
Thyroid disease (*n*, %)	12 (13.2)
Allergies (*n*, %)	32 (35.1)
Asthma/COPD (*n*, %)	2 (2.2)
Tumor other site (*n*, %)	22 (24.2)

CAD, coronary artery disease; BMI, body mass index; RT, radiotherapy; TIA, transient ischemic attack; PCI, percutaneous coronary intervention; CABG, coronary artery bypass graft; COPD, chronic obstructive pulmonary disease.

**Table 2:** Clinical history and CV risk factors grouping analysis.

Personal risk factors	All population	0–2 risk factors	≥3 risk factors (3–5)	*p*-value
	*N* = 91	*N* = 55 (60.4%)	*N* = 36 (39.6%)	
Age, years	56.85 ± 6.85	55.62 ± 6.68	58.72 ± 6.77	0.0337
Breast cancer side, *n* (%)				
Right	40 (43.96%)	27 (49.1%)	13 (36.1%)	0.2225
Left	51 (56.04%)	28 (50.9%)	23 (63.9%)	
Familiarity, *n* (%)	37 (40.66%)	17 (30.9%)	20 (55.6%)	0.0193
Smoke, *n* (%)				
0	62 (68.13%)	51 (92.7%)	11 (30.6%)	0.0000
1	9 (9.89%)	2 (3.6%)	7 (19.4%)	
2	20 (21.98%)	2 (3.6%)	18 (50%)	
Alcohol, *n* (%)	1 (1.1%)	0 (0%)	1 (2.8%)	0.3956
Physical activity, *n* (%)	42 (46.15%)	30 (54.5%)	12 (33.3%)	0.0472
Family history				
Premature CV death, *n* (%)	2 (2.2%)	1 (1.8%)	1 (2.8%)	1.0000
CHD, *n* (%)	8 (8.79%)	2 (3.6%)	6 (16.7%)	0.0541
CMP, *n* (%)	0 (0%)	0 (0%)	0 (0%)	1.0000
PVD, *n* (%)	1 (1.1%)	0 (0%)	1 (2.8%)	0.3956
Ictus, *n* (%)	1 (1.1%)	0 (0%)	1 (2.8%)	0.3956
Hypertension, *n* (%)	22 (24.18%)	5 (9.1%)	17 (47.2%)	0.0000
Diabetes, *n* (%)	4 (4.4%)	0 (0%)	4 (11.1%)	0.0220
Dyslipidemia, *n* (%)	33 (36.26%)	13 (23.6%)	20 (55.6%)	0.0020
Personal history	All population	0–2 risk factors	≥3 risk factors (3–5)	*p*-value
Sum risk factors	*N* = 91	*N* = 55 (60.4%)	*N* = 36 (39.6%)	
Sum risk factors, *n* (%)				
0	9 (9.89%)	9 (16.4%)	0 (0%)	0.0000
1	26 (28.57%)	26 (47.3%)	0 (0%)	
2	20 (21.98%)	20 (36.4%)	0 (0%)	
3	20 (21.98%)	0 (0%)	20 (55.6%)	
4	11 (12.09%)	0 (0%)	11 (30.6%)	
5	5 (5.49%)	0 (0%)	5 (13.9%)	
Weight, kg	65.27 ± 11.67	64.76 ± 11.42	66.06 ± 12.17	0.6085
Height, cm	163.89 ± 6.08	165.22 ± 5.8	161.86 ± 6.02	0.0093
BMI, kg/m^2^	24.32 ± 4.36	23.71 ± 3.92	25.24 ± 4.87	0.1017
Pregnancy, *n* (%)				
0	25 (27.47%)	18 (32.7%)	7 (19.4%)	0.4354
1	28 (30.77%)	14 (25.5%)	14 (38.9%)	
2	28 (30.77%)	17 (30.9%)	11 (30.6%)	
3	10 (10.99%)	6 (10.9%)	4 (11.1%)	
Preterm births, *n* (%)				
0	85 (93.41%)	52 (94.5%)	33 (91.7%)	0.6359
1	5 (5.49%)	2 (3.6%)	3 (8.3%)	
3	1 (1.1%)	1 (1.8%)	0 (0%)	
Spontaneous abortion, *n* (%)				
0	80 (87.91%)	50 (90.9%)	30 (83.3%)	0.4029
1	8 (8.79%)	4 (7.3%)	4 (11.1%)	
2	1 (1.1%)	0 (0%)	1 (2.8%)	
3	1 (1.1%)	1 (1.8%)	0 (0%)	
9	1 (1.1%)	0 (0%)	1 (2.8%)	
Therapeutic abortion, *n* (%)	0 (0%)	0 (0%)	0 (0%)	1.0000
Diabetes during pregnancy, *n* (%)	2 (2.2%)	1 (1.8%)	1 (2.8%)	1.0000
Hypertension during pregnancy, *n* (%)	1 (1.1%)	0 (0%)	1 (2.8%)	0.3956
Eclampsia, *n* (%)	0 (0%)	0 (0%)	0 (0%)	1.0000
Early menopause (<45 years), *n* (%)	25 (27.47%)	16 (29.1%)	9 (25%)	0.6690
Menopause, *n* (%)				
0	6 (6.59%)	4 (7.3%)	2 (5.6%)	0.6356
1	84 (92.31%)	51 (92.7%)	33 (91.7%)	
2	1 (1.1%)	0 (0%)	1 (2.8%)	
Menopause age, years	47.99 ± 5.23	48.11 ± 5.06	47.81 ± 5.56	0.7879
Cause of menopause, *n* (%)				
Surgical	7 (7.78%)	6 (11.1%)	1 (2.8%)	0.0642
Drug	59 (65.56%)	38 (70.4%)	21 (58.3%)	
Natural	24 (26.67%)	10 (18.5%)	14 (38.9%)	
Hormone replacement therapy, *n* (%)	1 (1.12%)	0 (0%)	1 (2.8%)	0.3956
Clinical characteristics				
Arrhythmias, *n* (%)	4 (4.4%)	1 (1.8%)	3 (8.3%)	0.2966
Known CAD (prior PCI or CABG), *n* (%)	0 (0%)	0 (0%)	0 (0%)	1.0000
CMPD/heart failure, *n* (%)	0 (0%)	0 (0%)	0 (0%)	1.0000
Pericarditis/myocarditis, *n* (%)	0 (0%)	0 (0%)	0 (0%)	1.0000
Allergies, *n* (%)	32 (35.16%)	15 (27.3%)	17 (47.2%)	0.0513
Polycystic ovary, *n* (%)	0 (0%)	0 (0%)	0 (0%)	1.0000
Cancer (other site), *n* (%)	22 (24.18%)	14 (25.5%)	8 (22.2%)	0.7247
Gastric ulcer, *n* (%)	0 (0%)	0 (0%)	0 (0%)	1.0000
Hiatal hernia, *n* (%)	4 (4.4%)	0 (0%)	4 (11.1%)	0.0220
Basal hyperglycemia, *n* (%)	0 (0%)	0 (0%)	0 (0%)	1.0000
NIDDM, *n* (%)	0 (0%)	0 (0%)	0 (0%)	1.0000
IDDM, *n* (%)	0 (0%)	0 (0%)	0 (0%)	1.0000
Hyperuricemia, *n* (%)	1 (1.1%)	0 (0%)	1 (2.8%)	0.3956
Hypertension, *n* (%)	4 (4.4%)	2 (3.6%)	2 (5.6%)	0.6466
Hypercholesterolemia, *n* (%)	15 (21.74%)	6 (14%)	9 (34.6%)	0.0438

RT, radiation therapy; CV, cardiovascular; CHD, cardiac heart disease; CMP, cardiomyopathy; PVD, peripheral vascular disease; BMI, body mass index; CAD, coronary artery disease; PCI, percutaneous coronary intervention; CABG, coronary artery bypass graft; NIDDM, non-insulin-dependent diabetes mellitus; IDDM, insulin-dependent diabetes Mellitus.

Data obtained were valuable in 91 out of 92 patients (severe artifacts during CT acquisition were present in one case), showing an overall incidence of 36.2% of pathologic Agatston calcium score. Specifically, the CAC score was 0 in 58 patients (63%), between 1 and 199 in 25 patients (27%), between 100 and 299 in 4 patients (4.3%), and >300 in 4 patients (4.3%). No differences were found according to the irradiated breast side (left vs. right) (Table 3).

**Table 3:** Dosimetric data and coronary artery exposure.

Dosimetric values*, average (SD)	Left-sided BC (*N* = 49, 55.1%)	Right-sided BC (*N* = 40, 44.9%)
Whole heart		
Dmean (Gy)	1.801 (0.770)	0.751 (0.621)
Left ventricle		
Dmean (Gy)	3.090 (1.805)	0.342 (0.218)
V5Gy (%)	10.25 (7.95)	0.000 (0.000)
V23Gy (%)	3.77 (3.54)	0.000 (0.000)
LAD		
Dmean (Gy)	7.854 (5.335)	0.592 (0.512)
V30 Gy (%)	11.46 (12.72)	0.000 (0.000)
V40 Gy (%)	3.37 (7.21)	0.000 (0.000)
LCx		
Dmean (Gy)	0.665 (0.513)	0.442 (0.452)
Dmax (Gy)	1.244 (1.137)	0.683 (0.701)
RCA		
Dmean (Gy)	0.518 (0.188)	1.179 (1.010)
Dmax (Gy)	0.889 (0.394)	2.086 (1.673)

VXGy, percentage of volume of a structure receiving X Gy; Dmax, maximum dose; D mean, mean dose; Gy = Gray; LAD, left anterior descending artery; LCx, left circumflex artery; RCA, right coronary artery.

* Dosimetric values available for 89 out of 92 patients.

Coronary calcifications distribution involved mainly the proximal-mid segments of major epicardial vessels.

The overall average of the mean doses to the cardiac structures during breast cancer RT is reported in Table 4.

**Table 4:** Dosimetric data and calcium scores.

Dosimetric data	All pop	0–2 risk factors	3–5 risk factors	*p-*value
	*N* = 91	*N* = 55 (60.4%)	*N* = 36 (39.6%)	
Dmean heart (Gy)	1.03 (0.64;2.14)	0.9 (0.56;1.66)	1.52 (0.71;2.34)	0.0280
Dmean left ventriculus (Gy)	1.16 (0.29;3.3)	0.59 (0.27;2.61)	1.85 (0.36;4.48)	0.0608
V5 left ventriculus (%)	1.1 (0;10.04)	0 (0;8.37)	3.05 (0;15.6)	0.0470
V23 left ventriculus (%)	0.01 (0;3.79)	0 (0;1.29)	0.63 (0;6.02)	0.0601
Dmean_LAD (Gy)	1.55 (0.54;8.16)	1.1 (0.47;6.08)	3.91 (0.6;9.45)	0.0859
V30 LAD (%)	0 (0;6.79)	0 (0;3.31)	1.34 (0;14.41)	0.0647
V40 LAD (%)	0 (0;0.01)	0 (0;0)	0 (0;0.27)	0.1569
Dmean circumflex artery (Gy)	0.46 (0.32;0.63)	0.41 (0.31;0.54)	0.56 (0.36;0.73)	0.0246
Dmax circumflex artery (Gy)	0.75 (0.52;0.99)	0.64 (0.47;0.84)	0.89 (0.57;1.17)	0.0413
Dmean coronary artery (Gy)	0.65 (0.44;0.93)	0.65 (0.4;0.95)	0.63 (0.5;0.85)	0.5430
Dmax coronary artery (Gy)	1.08 (0.74;1.74)	1.15 (0.71;1.82)	1.06 (0.79;1.65)	0.5764
Calcium score				
CAC (Agatston unit)	0 (0;18)	0 (0;0)	1.5 (0;54.5)	0.0013
Number of segments	0 (0;1)	0 (0;0)	0 (0;2)	0.0275
mAs	199 (199;200)	199 (199;200)	199 (199;200)	0.1720
DLP (mGy/cm)	42.98 (42.04;45.17)	43.04 (42.18;44.28)	42.88 (41.93;46.48)	0.9879
CAC-DRS 0 (Agatston 0)	54 (59.34%)	39 (70.9%)	15 (41.7%)	0.0055
CAC-DRS 1 (Agatston 1–99)	25 (27.47%)	13 (23.6%)	12 (33.3%)	0.3109
CAC-DRS 3 (Agatston 100–299)	4 (4.4%)	1 (1.8%)	3 (8.3%)	0.2966
CAC-DRS 4 (Agatston ≥300)	4 (4.4%)	0 (0%)	4 (11.1%)	0.0220
Left main	7 (7.69%)	1 (1.8%)	6 (16.7%)	0.0143
Proximal left anterior descending	25 (27.47%)	10 (18.2%)	15 (41.7%)	0.0141
Mid left anterior descending	14 (15.38%)	7 (12.7%)	7 (19.4%)	0.3852
Distal left anterior descending	0 (0%)	0 (0%)	0 (0%)	1.0000
First diagonal	5 (5.49%)	2 (3.6%)	3 (8.3%)	0.3806
Second diagonal	1 (1.1%)	0 (0%)	1 (2.8%)	0.3956
Proximal circumflex	11 (12.09%)	4 (7.3%)	7 (19.4%)	0.1050
Obtuse marginal	3 (3.3%)	1 (1.8%)	2 (5.6%)	0.5600
Proximal right	5 (5.49%)	0 (0%)	5 (13.9%)	0.0081
Mid right	7 (7.69%)	3 (5.5%)	4 (11.1%)	0.4284
Distal right	2 (2.2%)	1 (1.8%)	1 (2.8%)	1.0000
Posterior descending	1 (1.1%)	0 (0%)	1 (2.8%)	0.3956
Posterolateral	0 (0%)	0 (0%)	0 (0%)	1.0000
Number of vessels = 1	16 (17.58%)	8 (14.5%)	8 (22.2%)	0.3469
Number of vessels = 2	10 (10.99%)	5 (9.1%)	5 (13.9%)	0.5089
Number of vessels = 3	2 (2.2%)	0 (0%)	2 (5.6%)	0.1539
Number of vessels = 4	4 (4.4%)	1 (1.8%)	3 (8.3%)	0.2966
kv				
100	4 (4.44%)	4 (7.3%)	0 (0%)	0.1540
120	86 (95.56%)	51 (92.7%)	35 (100%)	
Slice thickness				
0.6 mm	3 (3.3%)	1 (1.8%)	2 (5.6%)	0.2215
0.625 mm	9 (9.89%)	4 (7.3%)	5 (13.9%)	
1.25 mm	1 (1.1%)	0 (0%)	1 (2.8%)	
2.5 mm	78 (85.71%)	50 (90.9%)	28 (77.8%)	

Dmean, dose mean; DLP, dose–length product; LAD, left anterior descending artery; CAC, coronary artery calcium; CAC-DRS, coronary artery calcium data and reporting system.

We included age and laterality as a covariate in the multivariable regression models to account for their potential confounding effect. The analyses are detailed in Supplementary Table S1.

A univariable correlation analysis (Spearman's rho) between radiation dose and CAC score was performed (Supplementary Table S2) with no additional substantial information, thus confirming the associative and not causal role of radiation Tx on CAC score.

Neither the amount of incidental dose given to any cardiac structure nor the side of the irradiated breast has demonstrated an association with the CAC score observed.

In the segmental analysis, pathologic CAC scores were observed particularly in patients with known hypertension and high cholesterol levels and those who were active smokers at the time they received RT. Additionally, after dividing our patients cohort into two groups according to the total number of CVRF [Group 1, *n* = 55 (60.4%): 0–2 CV risk factors; Group 2, *n* = 36 (39.6%): 3–5 CV risk factors], significant differences were observed in mean radiation dose to the heart (*p* = 0.02), the percentage of the left ventricle volume receiving a 5 Gy radiation dose (*p* = 0.04) and mean dose delivered to the left circumflex artery (*p* = 0.02).

**Figure 2:**
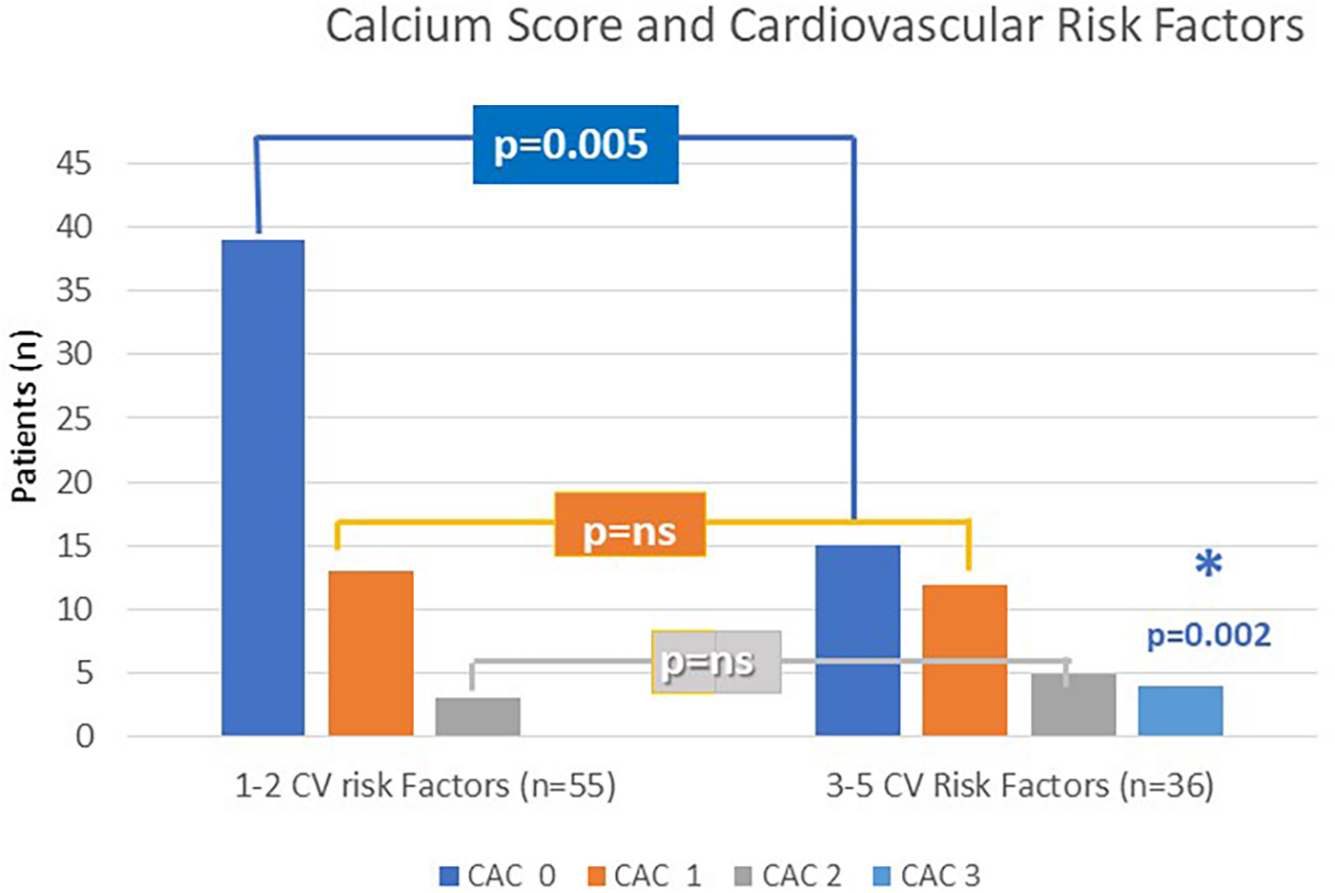
CAC score according to CV risk factors grouping (Group 1 and Group 2).

Normal CAC scores (CAC-DRS 0; Agatston 0) were recorded in 70.9% of Group 1 vs. 41.7% of Group 2 (*p* = 0.005), while CAC-DRS 3 (Agatston > 300) was observed in 11.1% of Group 2 exclusively (*p* = 0.02) (Figure 2). The association between CAC score and risk-factor grouping remained significant after adjusting for radiation dose delivered, age, and laterality (*p* = 0.023). These Group 2 patients with high CAC scores (*n* = 8), accounting for 8.8% of the enrolled population, underwent further investigation with first- and second-level diagnostic evaluations. They presented with a clinical equivalent of silent ischemia (*n* = 2) on a stress test, stable angina on effort (*n* = 1), and critical left anterior descending artery stenosis (*n* = 2) detected with contrast CT scan, requiring percutaneous revascularization. In the remaining three cases, coronary CT scan showed mild to moderate coronary disease requiring preventive drug therapy. No MACCE including myocardial infarction, stroke, and cardiovascular death occurred among our patient population.

As expected, the risk of higher CAC scores was associated with increased age (*p* < 0.0001), early menopause (*p* = 0.01), prolonged follow-up underlying hypertension (*p* = 0.01), high cholesterol levels (*p* = 0.007), and smoking habits (*p* = 0.04) at the time of RT and the sum of multiple risk factors (0.001).

## Discussion

Breast cancer-specific mortality has been progressively reduced over the past 30 years, due to improvement in oncologic therapies and more extensive screening allowing for earlier disease detection (15, 16). This, in combination with a slight increase in breast cancer incidence rate per year (17), has resulted in a growing number of long-term survivors who may be potentially exposed to causes of death unrelated to breast cancer (18). Consequently, the prevention of breast cancer treatment-induced complications, such as cardiovascular diseases (CVDs), is a definite need. CVD is the leading cause of death in women worldwide, and in patients with breast cancer, it is also an important cause of mortality (19).

Colzani et al. (20) showed that in patients with breast cancer, 12% of all deaths within 10 years after diagnosis were attributed to CVDs, and among elderly patients (>65 years), 24% of deaths were CVD-related.

Over the last two decades, more in-depth knowledge and a better understanding of the mechanisms of interaction of oncological therapies and their potential impact on patients' quality of life have raised awareness and created concern about the potential increase in the risk of CVD, especially in patients with pre-existing risk factors (21).

Most breast cancer treatment guidelines and survival prediction scores mainly focus on tumour/antigenic characteristics, while patients’ risk factors are hardly taken into account.

The coronary atherosclerotic plaque has calcium as an essential component. Detection of CAC on a chest CT scan has proven to be a strong and predictable marker of future cardiac events and obstructive coronary disease in the general population (6). In a recent study, Brix et al. (22) demonstrated how it is possible to use CAC scoring to downgrade or upgrade the risk of obstructive CAD in asymptomatic patients.

Additionally, it is well known that CAC increases the risk of cardiovascular events by several fold and high CAC scores increase risk and add incremental prognostic information to traditional risk factors (23). Although there is a moderate correlation between the number of risk factors for atherosclerosis and CAC, the prognosis of patients with no CAC has consistently been shown to be excellent for both the risk of mortality and cardiovascular events.

A 10-fold increase in CVD risk has been associated with a CAC score above 100 in several studies. The recently published CONFIRM trial demonstrated that high CAC scores in patients without previous atherosclerotic cardiac disease may be considered equivalent, in terms of cardiovascular risk, to stable secondary prevention populations, thus suggesting the need for more broadly targeted preventive approaches (24).

Following current preventive medicine guidelines, in a large population-based imaging study, Ties et al. (25) found that conventional risk scores failed to detect the majority of subjects with a very high CV risk profile, as indicated by a CACS ≥ 300 and CACS ≥100. The findings of this study suggest that many individuals with high CACSs are actually left unidentified and untreated.

So far, no randomized trials have been conducted on the effectiveness of calcium score-based treatment strategies aimed at cardiac disease prevention and mortality reduction (26). The Risk Or Benefit IN Screening for CArdiovascular diseases (ROBINSCA) trial is the first randomized controlled trial conducted in 12,950 potentially high-risk women and men investigating the value of CAC imaging followed by preventive treatment in reducing coronary heart disease-related mortality and morbidity (27). Age, high waist circumference, family history of cardiac heart disease (CHD), smoking at baseline, diabetes mellitus, known hypertension, and hypercholesterolemia at baseline were identified as predictors in the backward regression analysis of the presence of CAC and CAC of 400 or greater in women. High CAC is a rare finding in younger women but becomes more common with advancing age, especially post-menopause, when the protective effects of estrogen diminish. This variation emphasizes the importance of sex-specific considerations in evaluating patients, according to CV traditional risk factors, as highlighted in the current study.

The understanding of these findings hinges on CAC, which reflects the cumulative lifetime effect of modifiable and non-modifiable risk factors on vulnerable tissue, and has emerged as an excellent tool to improve CAD risk stratification (28).

Radiation therapy exposure is an additional external and non-modifiable factor that may play a negative inflammatory role in the coronary endothelial and microvascular network.

The pathophysiology of radiation-induced coronary artery disease (CAD) is remarkably complex, causing both microvascular and macrovascular damage in coronary arteries (15). Plaque formation in radiation-induced CAD is thought to mimic spontaneous atherosclerosis that, when combined with traditional risk factors for atherosclerotic plaque development, leads to the accelerated development of obstructive CAD observed in this patient population.

In a study of 59,502 asymptomatic healthy subjects aged 40–75 years (mean age 54 ± 8), the median coronary artery calcium score for individuals aged 40–54 years was 0 Agatston units (29).

Similarly in the current study, despite the patients having received RT, coronary CT calcium score analysis showed a median CAC of 0 Agatston units in subjects aged <54 years and a mean CAC score of 57 ± 138 Agatston units (range 3–690) in those aged 55–65 years, comparable to the calcium score observed in older (aged 65–75 years) healthy non-irradiated subjects.

Darby et al. (6) in their analysis of 2,168 Nordic breast cancer patients described a significant excess relative risk associated with radiotherapy. The average estimated mean radiation dose to the heart was 4.9 Gy overall. For every increase of 1 Gy in the mean dose to the heart, the rate of major coronary events rose by 7.4% (95% CI: 2.9–14.5; *P* < 0.001). When categorized by mean radiation dose to the heart—under 2, 2–4, 5–9, or 10 Gy and above—the percentage increases in major coronary events compared with an estimated rate of zero cardiac dose were 10%, 30%, 40%, and 116%, respectively. Although Darby et al. did not find a threshold below which there was no risk, the results of a meta-analysis performed on 451,386 patients by Little et al. (30) supported the statement from the Health Protection Agency's AGIR that a significantly elevated risk was detectable only for exposures above approximately 0.5 Gy.

In recent years, the importance of dose to cardiac substructures has emerged concerning their contribution to the development of cardiovascular diseases. With the recognition that radiation dose is associated with an increased frequency of cardiovascular damage, awareness has grown of the need to minimize the dose to all cardiac segments and structures (31, 32).

Advancements in radiation techniques have enabled the development of the concept of high-precision radiotherapy. The ability to obtain precise dose calculations for structures and organs at risk, along with improved radiobiological understanding of the interactions between radiation and healthy tissues, has allowed for the definition of dose constraints associated with a low risk of complications. Techniques focused on reducing the dose to the heart and coronary arteries include intensity-modulated radiotherapy, deep inspiration breath-hold (33), the prone position (34) (although not always optimal for cardiac sparing), and the use of proton therapy, which is available in highly specialized centers (8). However, for each woman, the benefits need to be balanced against the risks. Therefore, the benefit provided by advanced heart-sparing radiotherapy through reduced cardiac exposure is evident across all patients, but it may be even more pronounced in those with higher 10-year baseline cardiovascular risk scores. In the current study, abnormal CAC scores helped identify the most vulnerable subgroup.

In the SAVE-HEART Study (35), a dosimetric analysis indicated a higher excess relative risk of cardiovascular events with free-breathing compared with deep inspiration breath-hold, resulting in a 64.7% relative increase in cardiac risk. In the study by Figlia et al. (32), the use of intensity-modulated radiotherapy for locoregional treatment, by reducing the mean cardiac dose, was associated with a 10-year excess absolute risk reduction of 6% for ischemic heart disease compared with 3D conformal radiotherapy in elderly patients with high cardiovascular risk.

The current study reaffirmed the association between cardiac events and established risk factors such as early menopause, hypertension, hypercholesterolemia, and smoking. In the current study, the presence of 3–5 cardiovascular factors increased the dose to the cardiac structures, potentially exposing this group of patients to a higher risk of cardiac diseases. Heart-sparing strategies should incorporate systematic cardiovascular risk assessment prior to radiotherapy to identify the most vulnerable patients and to develop comprehensive risk-reduction plans that integrate all aspects of oncologic care.

In a systematic review of all breast cancer RT dosimetric reports involving 40,781 women whose smoking habits were known, Taylor et al. (36) estimated an excess rate ratio (ERR) of 0.04 per Gy of whole-heart dose. Since the risk of dying from heart disease before age 80 was estimated at 1.8% for non-smokers and 8.0% for smokers, based on the 2010 female death statistics from Western EU countries, delivering a 4 Gy mean heart dose increases these risks by 1.16 times, corresponding to an absolute increase in cardiac mortality of 1.2% for smokers.

Among traditional risk factors, although some results have indicated that the risk of CVD is more pronounced in hypertensive women than in men, overall, the cardiovascular risk associated with high blood pressure does not seem to differ by gender (37). The incidence of diabetes is equivalent in women and men; however, the relative risk for diabetes-induced CVDs is higher in women. Two different meta-analyses conducted on studies of Type 2 and Type 1 diabetes demonstrated a significantly higher excess risk of all-cause mortality and the incidence of fatal and non-fatal cardiac events in women (38, 39). Regarding cholesterol, women generally have a more favorable lipid profile before menopause, which can be attributed to the protective effects of estrogen. After menopause, increases in LDL cholesterol and decreases in HDL cholesterol are common, leading to a rise in CVD risk that greatly exceeds the risk in men of the same age (40). A high cardiovascular risk profile has been detected in women who smoke. In fact, smoking inhibits the cardiovascular-protective effects of estrogen or its production in women (41), potentially leading to more significant arterial damage and a higher risk of atherosclerosis.

The data found in this study suggest that pre-existing CV risk factors at the time of RT treatment may serve as markers of the strong negative synergistic effect between modifiable CV risk factors and radiation exposure, predicting future coronary calcifications and cardiovascular events. However, the lack of a control group of non-irradiated patients or a cohort with zero CVRF does not allow for full separation of the effects of radiotherapy from baseline cardiovascular risk or natural aging. Therefore, future matched cohort studies are needed to better isolate the role of RT on long-term coronary changes. To achieve an effective CVD prevention in women undergoing RT for breast cancer, the assessment of traditional risk factors is paramount. A thorough understanding of the disease is essential for accurate diagnosis, treatment, and prevention of the additional negative effects of radiation therapy on coronary artery integrity and health, requiring systematic collection of information to be integrated into standard medical practices before RT (42).

In the current era, where the field of medicine is defined by precision and personalization, a simple, baseline pre-screening analysis based on the assessment of the strongest independent cardiovascular disease (CVD) risk factors and the assessment of coronary calcifications (CAC) on a baseline coronary CT scan could be a useful strategy for a prestine detection of high-risk individuals. Regarding radiotherapy, advancements in radiation techniques, along with improved radiobiological understanding of the interactions between radiation and healthy tissues, have allowed for the definition of dose constraints associated with a low risk of complications. Techniques focused on reducing the dose to the heart and coronary arteries are easily implementable and are part of the clinical practice of all radiotherapy centers (43). Taking into account patients' CVD risk factors in treatment decisions could help select the most appropriate balance between cancer treatment and the prevention of cardiac events.

There are some **limitations to the study**. The results apply to a White Caucasian population and cannot be directly extrapolated to all other racial groups. Most of the cardiac structures were outlined for the purpose of the study, except for the whole heart and, for some subjects, the left anterior descending artery, which made it impossible to optimize the treatment plan at the time of RT according to specific constraints. Nevertheless, the dosimetric constraints calculated for this study showed a high level of adherence to the most commonly used dosimetric constraints described in the DEGRO recommendations (Duma-DEGRO) (42). It should be pointed out that the dose metrics referred to moderate hypofractionation, actually the new standard for breast cancer RT. The cardiac structures were delineated using the non-contrast-enhanced CT simulation. This posed some difficulties in visualizing the entire trajectory of the coronary arteries, particularly the circumflex and right coronary arteries; therefore, in some areas, the anatomical region where they should have been located was outlined. For the same reason, to avoid approximations, it was considered appropriate not to proceed with further segmentation of the coronary arteries into proximal, mid, and distal sections. The study lacks a control group of non-irradiated patients or a cohort with zero CVRF; therefore, these data do not allow for full separation of the effects of radiotherapy from baseline cardiovascular risk or natural aging. The mean follow-up of the present study was 8.8 (±2) years, representing an intermediate time point in the evaluation of cardiovascular risk, which increases over time, even beyond 30 years (6).

## Data Availability

The datasets presented in this study can be found in online repositories. The names of the repository/repositories and accession number(s) can be found below: https://www.zenodo.org. Zenodo respository: 10.5281/zenodo.13373922.
