# Supplementary material for: Coronary artery calcium and cardiovascular risk factors analysis after radiotherapy for breast cancer (the CLARIFIER: a gender-based preventive medicine study)
Source: Front Cardiovasc Med. 2025 Aug 4;12:1615793. doi: 10.3389/fcvm.2025.1615793 (PMC12359178; doi:10.3389/fcvm.2025.1615793)

**Supplementary Table 1:** Results of the multiple regression analysis, including both unstandardized and standardized beta (β) coefficients, along with their 95% confidence intervals and p-values


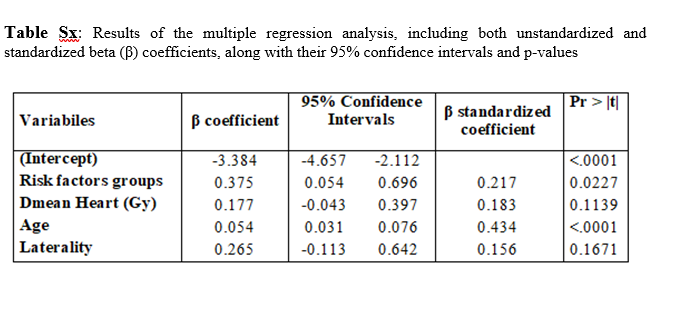

Supplement: Supplementary file 1 [file Datasheet1.zip › Table 1.docx]
